# F251. REVISITING THE RELATIONSHIP BETWEEN NEUROCOGNITION AND SUBJECTIVE QUALITY OF LIFE IN SCHIZOPHRENIA

**DOI:** 10.1093/schbul/sby017.782

**Published:** 2018-04-01

**Authors:** Eric Tan, Stuart Lee, Susan Rossell

**Affiliations:** 1 Swinburne University; 2 Monash Alfred Psychiatry Research Centre, The Alfred Hospital and Monash University, Central Clinical School; 3 Monash Alfred Psychiatry Research Centre, The Alfred Hospital and Monash University, Central Clinical School, Swinburne University of Technology, The University of Melbourne, St. Vincent’s Hospital

## Abstract

**Background:**

Neurocognitive impairments are a major feature of schizophrenia and present long-term challenges to the quality of life (QOL) of patients. Their contribution to a patient’s life satisfaction (subjective QOL; sQOL) has been much investigated, however, results have been equivocal and often non-significant. This contrasts with relatively more evidence for the neurocognition-objective QOL (oQOL) relationship. Previous work has also not investigated any differences in the subjective QOL associations between lower-order (e.g. processing speed, attention) and higher order (e.g. executive function) cognitive abilities. This study sought to better characterise the neurocognition-sQOL relationship through 3 separate analyses in clinical and healthy control samples: 1) examining correlational relationships between oQOL and sQOL and both lower-order and executive cognitive skills; 2) examining if lower-order or executive cognitive skills moderate the relationship between oQOL and sQOL; and 3) examining if the relationship between sQOL and both lower-order and executive cognitive skills differs between groups.

**Methods:**

Data from 57 schizophrenia/schizoaffective disorder patients (age: M=43.40, SD=10.85) and 48 healthy controls (age: M=39.82, SD=13.89) was analysed. QOL was assessed using the Lehman’s Quality of Life Interview. Lower-order cognitive skills were assessed using 9 tasks: Trail Making Test-A, symbol coding, animal fluency, spatial span, letter-number span, continuous performance test, Hopkins verbal learning test, brief visuospatial memory test and digit span. Executive function was measured via: Mazes, MSCEIT-ME, DKEFS Colour-Word Interference Test (Inhibition and Switching) and letter fluency. All task scores were converted to z-scores and composites were calculated to represent lower-order cognition and executive function.

**Results:**

In line with the literature, the results revealed significant correlations between oQOL and sQOL but no associations between sQOL and either cognition measure in both groups (Analysis 1). In Analysis 2, neither lower-order nor executive cognitive skills moderated the relationship between oQOL and sQOL in either patients or controls. In Analysis 3, group membership moderated the relationship between executive function and sQOL (p=.037), with a positive relationship for controls but negative relationship for patients. Group did not moderate the relationship between lower-order cognition and sQOL (p=.16).

**Discussion:**

The relationship between cognition and sQOL appears to be more related to higher-order abilities relating to idea generation, inhibition and reasoning than lower-level functions. Additionally, directional group differences in this relationship may reflect better executive functions leading to lower sQOL assessment in patients and thus lower ratings compared to controls who predictably rate higher.

